# An Autograph Letter of Hahnemann

**Published:** 1890-11

**Authors:** Samuel Hahnemann

**Affiliations:** Paris


					﻿tCrul	L&~.
'hZotr^*^
'j^&F ^f/vcj tn^tsrrz-cd/ej fre jet",
w
f&nrf vutie ifrnJetef-L&e ‘t/t/z£tot<jc,£(d/e!
e^ u>rt<° £>-fi/n.e Sct/nfi!• f^tnovtA,
S)e ^te^c SJt^e 3& j^o-c - 'mesn.e
Qwne. ^iA^e. t	partite'
/eU{^^ ■
/842>
'Scz/m.u.e/
KvT<iGiRKS^L Letter of Samuel Hahnemann.
				

